# Ethical and legal challenges of integrating artificial intelligence into paediatric surgery

**DOI:** 10.1308/rcsann.2025.0110

**Published:** 2026-02-23

**Authors:** A Anand, CK Sinha

**Affiliations:** ^1^ Monash Health, Clayton, Australia; ^2^ Portland Hospital, London, UK; ^3^ St George's Hospital, London, UK

**Keywords:** Artificial intelligence, Paediatric surgery, Liability, Ethics, Data protection

## Abstract

**Introduction:**

Artificial intelligence (AI) technologies are increasingly being trialled with applications spanning imaging, robotic assistance and early risk prediction. Children’s unique legal status presents distinct ethical and legal concerns. The objective of this review was to outline key ethical and legal challenges from AI integration into paediatric surgery and propose practical strategies for clinicians and policymakers.

**Methods:**

This article is a narrative review of peer-reviewed and grey literature (2015–2025), and statutory guidance (including the Information Commissioner’s Office [ICO] Children’s Code, Medicines and Healthcare products Regulatory Agency [MHRA] Software as a Medical Device Roadmap, General Medical Council guidance), and documentation from paediatric AI hubs. Ethical analysis was framed using principlism (autonomy, beneficence, non-maleficence, justice), with modifications for paediatric contexts (evolving capacity, best interests).

**Findings:**

Five core challenges were identified: safeguarding sensitive paediatric data; facilitating informed consent; mitigating algorithmic bias; clarifying liability in adaptive systems; and harmonising regulatory oversight. Recommendations include federated learning networks, transparent model cards, a pooled indemnity fund, AI literacy training and ongoing performance audits.

**Conclusions:**

AI holds promise for safer, more equitable paediatric surgery with robust ethical safeguards, updated legal frameworks and sustained regulatory vigilance. What is already known: (i) AI enhances image guidance, robotic precision and risk prediction in surgery; (ii) children’s data require special protection under the UK General Data Protection Regulation and ICO Children’s Code; and (iii) NHS England funds AI pilots, but paediatric-specific governance remains underdeveloped. This study: (i) maps UK-specific ethical (privacy, consent, bias) and legal (Montgomery, Consumer Protection Act 1987, MHRA) duties in AI-assisted paediatric surgery; (ii) proposes a federated data-sharing consortium and tiered liability model, and offers a practical governance framework for clinicians, regulators, and NHS boards.

## Introduction

In the UK, more than 85,000 paediatric surgeries are performed annually in the National Health Service (NHS).^1^ Since 2020, the NHS AI Lab has allocated more than £140 million to machine learning initiatives, including some focus on paediatrics.^2^ Paediatric artificial intelligence (AI) intersects with complex legal duties such as informed consent (*Montgomery v Lanarkshire*, 2015), heightened data privacy, and evolving child capacity.^3^ Adaptive algorithms that learn post-deployment blur the boundary between product and practice, complicating legal accountability.

Product vs practice denotes continuous-learning software that may change after first placement on the market, redistributing responsibility across manufacturer, clinician and provider organisation.^4,5^ When harm arises from an update implemented outside the approved change envelope, accountability rests with the manufacturer. The approved change envelope is the pre-agreed scope in which software updates may occur after market entry, with specified validation and post-market monitoring requirements.^6^ Routine recording of model version, update status and the clinical decision path (acceptance or override, with justification) enables clear attribution.^5^ This review synthesises emerging ethical and legal implications and outlines practical solutions for AI governance in paediatric surgery.

## Methods

We conducted a narrative review of peer-reviewed journals, grey literature, AI pilot documentation and professional guidelines published between 2015 and 2025.^7^ The ethical analysis used Beauchamp and Childress’s four-principles framework, with adaptations for child-specific issues such as best interests and Gillick competence.^8^ Legal analysis drew on case law, the Consumer Protection Act 1987, and recent Medicines and Healthcare products Regulatory Agency (MHRA) and Information Commissioner’s Office (ICO) guidance.^5,9^

## Findings and discussion

### Data privacy and security

In the UK, children’s health data are protected under the Data Protection Act 2018 and Article 9 of the UK General Data Protection Regulation, requiring a Data Protection Impact Assessment (DPIA).^10^ Operative imaging and video cannot be reliably anonymised, especially in cloud environments. True anonymisation removes any reasonable means of re-identification, whereas pseudonymisation merely replaces identifiers with codes and remains re-identifiable via a key.^11,12^ Operative media often contain direct and indirect identifiers such as faces and voices, timestamps, theatre layouts, device screens, rare anatomical variants and distinctive surgical instruments; cross-referencing with theatre schedules, Picture Archive and Communication System audit logs or publicly available media renders re-identification a credible risk.^13,14^ A November 2024 cyber-attack on Alder Hey Children’s NHS Foundation Trust exemplifies these risks.^15^

Despite key coding, such personal data must comply with Article 9 processing conditions, a DPIA and strict access controls and cross-border transfer requirements.^10^ Cloud workflows introduce residual risks from multitenant processing, background logging, back-up copies and derived data sets used for model training, which complicate retention and erasure. Breaches can have lifelong consequences for affected children. Ethical AI deployment mandates technical safeguards such as differential privacy, homomorphic encryption and secure enclaves to uphold fiduciary duties (Table 1).^13^

**Table 1:** Privacy-preserving safeguards^16^

Safeguard	Definition
Differential privacy	A mechanism that injects calibrated statistical noise so that inclusion or exclusion of any single record does not materially change released results, quantified by a stated privacy budget
Homomorphic encryption	A cryptographic scheme that permits specified computations on ciphertext, producing an encrypted result that decrypts to the correct plaintext output while the plaintext remains concealed throughout processing
Secure enclaves (confidential computing)	Hardware-based execution in a measured and attested isolated environment; data are decrypted only in the enclave and remain opaque to the host operating system and administrators
Federated learning	Distributed model training in which parameters are learned across participating sites while raw data remain local; only model updates are exchanged

### Informed consent and assent

*Montgomery v Lanarkshire* obliges clinicians to disclose risks material to a reasonable parent or Gillick-competent child.^17–19^ For AI-assisted tools, this includes the algorithm’s function, accuracy with uncertainty, autonomy level and known limitations.^20^ Families should be told that a human professional remains responsible for decisions and that declining the tool does not remove access to usual care.^20^ Where software is updated under an approved change plan, material changes that alter risk or capability should prompt renewed discussion and, when appropriate, refreshed consent.

Best practice includes:

- Provide a plain-English summary stating intended use, performance with uncertainty, limits, and oversight, supported by a judicious explanatory visual.^20^
- Record in the clinical note the model identity and version, the output presented, whether the output was accepted or overridden, and the clinical reasoning.^20^
- Offer dynamic consent options that allow families to adjust permissions for data use and sharing over time, with clear routes to withdraw.^9,20^
- Assess capacity and assent in line with Gillick principles, involve an interpreter or advocate where needed, and use accessible formats.^19,20^
- Explain data protection safeguards, including retention, access control and routes to correction.^5,13^
- Revisit consent if a substantive software update changes the risk profile or the way the tool is used in care.^5,13^

### Algorithmic bias

Non-representative training data can embed systemic biases, affecting treatment equity.^21^ Developers should: (i) report demographic breakdowns in training data sets; (ii) publish bias-testing results with predefined fairness thresholds; and (iii) disclose failure contexts and limitations.^21^

Transparency can be operationalised through model cards, a structured one- to two-page disclosure of intended use, data sources, evaluation setting, performance, including subgroup metrics and limitations.^22^ This enables providers to monitor fairness proactively.^21^

## Legal considerations

### Liability frameworks

Harm arising from surgical AI engages multiple legal regimes: (i) negligence (Bolam/Bolitho); (ii) informed consent (Montgomery); (iii) product liability (Consumer Protection Act 1987); and (iv) organisational duty (Health and Social Care Act 2012).^3,23^ Adaptive algorithms complicate these frameworks. The MHRA Software and AI as a Medical Device Change Programme Roadmap (2025) anticipates use of a predetermined change control plan and continuous post-market surveillance, including Yellow Card reporting.^24^

A tiered liability model is proposed: (i) clinicians retain primary responsibility; (ii) vendors assume liability for defective designs or updates; and (iii) a no-fault indemnity pool covers residual cases, mirroring NHS Resolution schemes.^5^

### Standard of care

Although Bolam/Bolitho remains the test, courts now consider Montgomery’s emphasis on patient autonomy.^17^ AI modifies expectations: (i) omission — failure to use validated tools may constitute negligence once superiority and endorsement are established; and (ii) blind reliance — uncritical acceptance of implausible outputs breaches duty.^7,20^

Clinicians should: (i) confirm algorithm version and validation status; (ii) cross-check AI outputs against clinical judgement; (iii) document the rationale for accepting or overriding recommendations; and (iv) maintain AI-related CPD (Table 2).^5,7,20^

**Table 2:** UK regulators and requirements for artificial intelligence used in paediatric surgery

Regulator	Definition	Requirements (sentences)
Medicines and Healthcare products Regulatory Agency	UK regulator for medicines and medical devices, including clinical software	The software must be classified and assigned a risk class. Any planned updates should be set out in an agreed change plan. Safety must be monitored after deployment. Incidents must be reported through Yellow Card, and corrective actions must be taken when required^5^
National Institute for Health and Care Excellence (NICE)	Appraises clinical and cost effectiveness, and issues guidance	Evidence must meet the NICE framework appropriate to the tool’s risk. Submissions should demonstrate clinical benefit and value for money. Analyses for children under 16 should be provided when relevant. Adoption recommendations should be followed once guidance is issued^25^
Information Commissioner’s Office	UK data protection regulator enforcing UK General Data Protection Regulation and the Children’s Code	A lawful basis and an Article 9 condition must be established for processing. A Data Protection Impact Assessment must be completed. Data must be minimised, secured and used transparently. Information for families must be clear, and services must meet the Children’s Code duties on best interests and age-appropriate design^10^

### Regulatory oversight

Software as a Medical Device (SaMD) refers to standalone software intended for one or more medical purposes and not part of a hardware device.^26^ In the UK, it is regulated as a medical device with the same obligations that apply to physical products.^4^ Clinical AI used for diagnosis, prediction, monitoring or treatment falls in this category and should be clearly identified as SaMD in clinical and procurement documentation. SaMD is risk-classified by intended purpose and potential harm in the same hierarchy as other devices (eg wound dressing class I; implantable pacemaker or defibrillator class III). Models that inform low-risk decisions may fall into lower classes, whereas those that drive or materially influence high-risk decisions are typically assigned to higher classes, with corresponding demands for evidence, post-market surveillance and incident reporting.^5,26^

The MHRA Software and AI as a Medical Device Change Programme Roadmap (2025) formalises this pathway by specifying classification, change control planning, proportionate evidence expectations and strengthened vigilance and Yellow Card reporting for software updates. Under the Yellow Card scheme, adverse incidents and performance problems involving SaMD must be reported; reports may be submitted by clinicians, provider organisations and manufacturers, and manufacturers have statutory timelines for serious incidents.^24^

Supplementary oversight is provided by the NHS AI Safety Institute and Care Quality Commission; cross-border tools must also comply with the European Union AI Act.^27^ A dedicated MHRA–ICO sandbox for paediatrics is anticipated.^5^

### Current clinical use of AI in paediatric surgery (UK)

In the NHS, use of AI in paediatric surgery is most evident in three areas. Preoperative planning uses three-dimensional segmentation, printing and virtual reality rehearsal for complex cardiac and craniofacial cases, improving operative understanding and family communication.^28^ Clinical documentation pilots introduce AI-enabled ambient scribing in outpatient settings to support consent discussions and reduce clerical load, under national implementation guidance.^13^ Remote collaboration platforms support intraoperative second opinions, training and service resilience across UK theatres, with published case experience from NHS sites.^29^ These deployments proceed within standard NHS governance, including data protection impact assessments and clinical safety cases, and align with national evaluation programmes such as the AI in Health and Care Award.^30^ Routine publication of model cards, systematic subgroup equity reporting and continuous post-deployment calibration remain variable and should be strengthened before wider-scale adoption.^22^

## Recommendations

### Transparency and explainability

Publish open-access model cards that state intended use, data sources, validation settings, performance, including subgroup results, limitations and update history. Make the model card available at the point of care and update it after any approved software change. Evidence of implementation is the presence of the current model card and a representative explanation figure in the clinical record.^10^

### Secure data infrastructure

Use Secure Data Environments so that raw data remain on site where feasible. Complete DPIAs and implement strong access control and encryption. Compliance is demonstrated by a signed DPIA and documented key-management and access logs.^30^

### Privacy-preserving learning

Prefer designs that minimise the movement of raw data. Apply federated learning, differential privacy and secure enclaves where proportionate. Report the privacy budget and any effect on accuracy in methods. Compliance is shown by protocol documents and model cards that report these parameters.^18,30,31^

### Clinical use and competence

Provide a yearly 4-hour AI safety module in the Intercollegiate Surgical Curriculum. The module should cover interpretation, recognition of failure modes, bias detection, documentation of accept or override decisions and escalation pathways. Completion rates and assessment scores confirm delivery.^7,20^

### Performance and equity monitoring

Stream the identified prediction and outcome pairs to monitoring dashboards that display accuracy, calibration and subgroup equity. Pre-specify thresholds that trigger pause, investigation and corrective action, and reinstate only after remediation. Audit trails and dashboard archives evidence adherence.^5,13^

### Liability, procurement and change control

State in contracts that day-to-day responsibility rests with the clinician, that responsibility transfers to the vendor when a design or update defect is demonstrated, and that residual edge cases are met by a no-fault indemnity pool. Require a predetermined change control plan and align local vigilance with Yellow Card reporting. Contract clauses and incident reports evidence compliance.^5,32^

## Conclusions

AI may revolutionise paediatric surgery, but its benefits depend on ethically and legally robust implementation. Privacy-first data design, child-centred consent, routine bias auditing and clear liability allocation will support responsible adoption. Coordinated oversight from MHRA, National Institute for Health and Care Excellence and ICO reinforced by real-world performance monitoring can maintain public trust while advancing surgical innovation.

## Competing interests

The author/s declare no competing interests.

## Funding

The author/s received no financial support for the research, authorship and/or publication of this article.

## Ethics approval statement

Ethical approval was not required for this work, which is a conceptual/legal-ethical analysis of the integration of artificial intelligence into paediatric surgery and did not involve the collection, analysis, or reporting of identifiable human or animal data. The author(s) obtained professional permission within their clinical practice setting to explore existing approaches and policies relating to AI integration.

## Consent to participate statement

Not applicable.

## Author contributions

**A Anand**: Conceptualisation, Data curation, Formal analysis, Investigation, Methodology. **CK Sinha**: Formal analysis, Methodology, Project administration.

## Artificial Intelligence

The author/s declare that no AI was used to conduct the study or prepare the manuscript.
